# *refnx*: neutron and X-ray reflectometry analysis in Python

**DOI:** 10.1107/S1600576718017296

**Published:** 2019-02-01

**Authors:** Andrew R. J. Nelson, Stuart W. Prescott

**Affiliations:** a ANSTO, Locked Bag 2001, Kirrawee DC, NSW 2232, Australia; b School of Chemical Engineering, University of New South Wales, Sydney, NSW 2052, Australia

**Keywords:** neutron reflectometry, X-ray reflectometry, Bayesian analysis, computer modelling, *refnx*

## Abstract

*refnx* is a model-based neutron and X-ray reflectometry data analysis package written in Python. It is cross platform and has been tested on Linux, macOS and Windows. Its graphical user interface is browser based, through a *Jupyter* notebook. Model construction is modular, being composed from a series of components that each describe a subset of the interface, parameterized in terms of physically relevant parameters (volume fraction of a polymer, lipid area per molecule *etc.*). The model and data are used to create an objective, which is used to calculate the residuals, log-likelihood and log-prior probabilities of the system. Objectives are combined to perform co-refinement of multiple data sets and mixed-area models. Prior knowledge of parameter values is encoded as probability distribution functions or bounds on all parameters in the system. Additional prior probability terms can be defined for sets of components, over and above those available from the parameters alone. Algebraic parameter constraints are available. The software offers a choice of fitting approaches, including least-squares (global and gradient-based optimizers) and a Bayesian approach using a Markov-chain Monte Carlo algorithm to investigate the posterior distribution of the model parameters. The Bayesian approach is useful for examining parameter covariances, model selection and variability in the resulting scattering length density profiles. The package is designed to facilitate reproducible research; its use in *Jupyter* notebooks, and subsequent distribution of those notebooks as supporting information, permits straightforward reproduction of analyses.

## Abstract 2

The *refnx* Python modules for neutron and X-ray reflectometry data analysis are introduced. A sample analysis illustrates a Bayesian approach using a Markov-chain Monte Carlo algorithm to understand the confidence in the fit parameters.

## 1. Introduction   

The use of specular X-ray and neutron reflectometry for the morphological characterization of thin films in the approximate size range 10–5000 Å has grown remarkably over the past few years (Wood & Clarke, 2017 ▸; Daillant & Gibaud, 2009 ▸). Most neutron and X-ray sources have instruments to perform reflectometry measurements, and there is an ongoing need for accessible software programs for users of those instruments to analyse their data in a straightforward fashion, including the co-refinement of multiple contrast data sets. Several programs are available for this purpose, with a variety of different features (Nelson, 2006 ▸; Bjorck & Andersson, 2007 ▸; Kienzle *et al.*, 2011 ▸; Gerelli, 2016 ▸; Hughes *et al.*, 2016 ▸). These programs typically create a model of the interface, and either incrementally refine the model against the data using least-squares methods or use Bayesian approaches (Sivia & Skilling, 2006 ▸; Kienzle *et al.*, 2011 ▸; Hogg *et al.*, 2010 ▸) to examine the posterior probability distribution of the parameters (*i.e.* the statistical variation of the parameters in a model).

Given the number of publications arising from the reflectometry technique, it is vital that both the experiments and analyses are reproducible. Reproducibility in research is an underlying principle of science, but unfortunately it is not always possible to reproduce the results of others (Stark, 2018 ▸), because there is frequently not enough information provided in journal articles to repeat the analyses. Even if the data sets and the software packages used to analyse them are supplied in supporting information (most often they are not), a comprehensive, ordered set of instructions or a codified workflow would need to be provided (Möller *et al.*, 2017 ▸). One example addressing this reproducibility issue is the set of guidelines from the small-angle scattering community for the deposition of data and and associated models (Trewhella *et al.*, 2017 ▸; Pauw, 2013 ▸).

Here, we outline a new reflectometry analysis package, *refnx* (version number 0.1 is used in this paper; Nelson & Prescott, 2018*b* ▸), that helps to address the reproducibility issue for the reflectometry community (we do not mean that other programs are irreproducible, rather that the information provided in journal articles is often lacking). It does this by creating a scripted analysis workflow that is readily deposited alongside the publication, such as we have done with this paper (see the supporting information). The *refnx* Python package is specifically designed for use in *Jupyter* notebooks (Kluyver *et al.*, 2016 ▸), which provide a literate programming environment that mixes executable code cells, rich documentation of the steps that were performed and the computational output. By including the analysis, as performed by the authors, in such a notebook, and appending it as supporting information along with the data, readers are empowered to replicate the exact data analysis and potentially extend the analysis, provided they have set up the same computing environment (Millman & Pérez, 2014 ▸). Setting up the computing environment is simplified using the *conda* package manager (Continuum Analytics, 2017 ▸) and an environment file, although other approaches are available.

## 2. Method   

**Figure 1:**
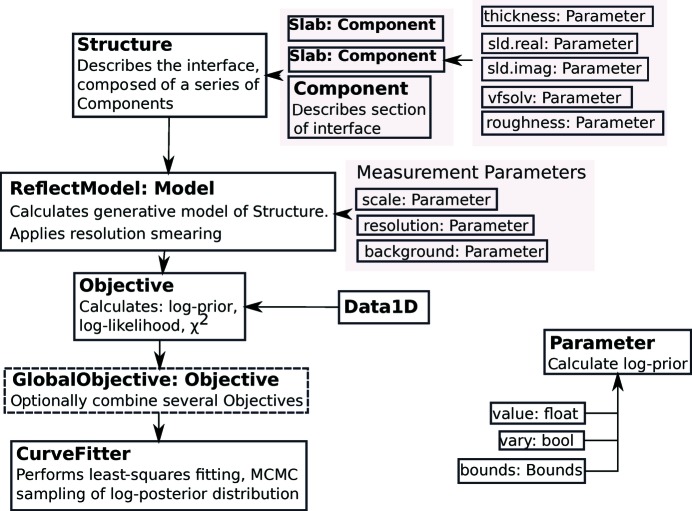
A schematic flow chart, showing the relationship between the classes that make up a typical reflectometry curve-fitting problem. The key step for the user is assembling materials (Component) such as a ‘Slab: Component’ (a Component that is a Slab) and encoding prior knowledge into each Parameter that describes that Component.

*refnx* is written in Python with an extensible object-oriented design (Fig. 1 ▸) in which the user creates a model of the sample based on what they know about its composition, with refinement of that model against the data. As with *Motofit* (Nelson, 2006 ▸), it calculates reflectivity using the Abeles method (Heavens, 1955 ▸) for specular reflection from a stratified medium. Detailed documentation for *refnx* is available online (https://refnx.readthedocs.io/) and is distributed with the package.

The building block of the analysis is the Parameter object, which represents a model value [*e.g.* the scattering length density (SLD) of the material], whether that value is allowed to vary in a fit, and a bounds attribute. The bounds are a probability distribution representing pre-existing knowledge of a parameter’s value, called a prior probability. A prior might be a simple uniform distribution that specifies a lower and upper bound (*e.g.* volume fraction is in the interval [0, 1]), or a normal distribution that represents an experimentally derived value and its associated uncertainty (*e.g.* thickness is 100 ± 4 Å). Any of the scipy.stats (Jones *et al.*, 2001 ▸) continuous distributions, or other distributions created by the user, can be used for this purpose. Algebraic relationships between Parameter objects can be applied to permit more sophisticated constraints that can cross between Component objects (*e.g.* the sum of the thicknesses of several layers is known to some uncertainty).

### 2.1. Structure representation   

The Structure object represents the interfacial model, assembled from individual Component objects in series. Each Component represents a subset of the interface and selected attributes of the Component can be described by physically relevant Parameter objects. The simplest and most familiar Component is a Slab, which has a uniform SLD, thickness, roughness and volume fraction of solvent. The simplest models are simply a series of Slab objects. More sophisticated components include LipidLeaflet (a lipid monolayer, or one-half of a lipid bilayer) and Spline (for free-form modelling of an SLD profile using spline interpolation). It is straightforward to develop/modify new components for different structural functionality, a consequence of the program design.

To include further prior knowledge of the real sample into the model, each Component can contribute to the prior probability in addition to its constituent Parameter objects. This is useful when a Component has a derived value, such as surface excess, which is already known.

To calculate the reflectivity from the series of Component objects that form the model, each Component has a *slabs* property that represents a discretized ‘slice’ approximation to a continuous SLD profile for its particular region of the interface. A Slab object has a single slice because it is a single thickness of uniform SLD. A LipidLeaflet is made of two slices (head/tail regions), but the Spline has many thin slices approximating the smooth curve. Each of these slices has uniform SLD, with the Névot–Croce approach being used to describe the roughness between them (Névot & Croce, 1980 ▸).

The Structure object is used to construct a ReflectModel object. This object is responsible for calculating the resolution-smeared reflectivity of the Structure, scaling the data and adding a *Q*-independent constant background [via the scale and background Parameter objects; *Q* = (4π/λ)sin(Ω), where Ω is the angle of incidence and λ is the wavelength of the incident radiation]. There are different types of smearing available: constant d*Q*/*Q*, point-by-point resolution smearing read from the data set of interest or via a smearing probability kernel of arbitrary shape (Nelson & Dewhurst, 2014 ▸). The constant d*Q*/*Q* and point-by-point smearing use Gaussian convolution, with d*Q* representing the full width at half-maximum (FWHM) of a Gaussian approximation to the instrument resolution function (van Well & Fredrikze, 2005 ▸).

### 2.2. Model/data comparison   

The Objective class is the comparator of the predicted and measured reflectivities, using the ReflectModel and a data set, Data1D, to calculate χ^2^, log-likelihood [equation (2)], log-prior, residuals and the generative model. The Data1D object has *x*, *x_err*, *y* and *y_err* attributes to represent *Q*, d*Q*, *R* and d*R*, respectively. As is standard for many reflectometry data files, the Data1D object reads a three- or four-column plain-text datafile. A three-column data set represents *Q* (Å^−1^), *R* and d*R* (one standard deviation). A four-column data set represents *Q* (Å^−1^), *R*, d*R* and d*Q* (Å^−1^). d*R* is the uncertainty in the reflectivity, *R*, and d*Q* specifies the FWHM of the instrument resolution function, for each data point. Extending Data1D would allow other formats to be read – at the moment there is no standardized data format for reflectometry. One example of this could be a wavelength-dispersive file using (Ω, λ) data instead of *Q*, such as that used in energy-scanned X-ray reflectometry, or sometimes produced by wavelength-dispersive neutron reflectometers. In such a case, ReflectModel could be subclassed to make full use of this energy-dispersive information. The creation of a standardized data format for reflectometry would facilitate ingestion of data and allow other important information, such as experimental metadata, to be used.

An Objective can be given a Transform object to permit fitting as log_10_ *R* versus *Q* or *RQ* ^4^ versus *Q*; the default (no Transform) is *R* versus *Q*. Several Objective objects can be combined to form a GlobalObjective for co-refinement. The object-oriented nature of the program allows reuse of Parameter and Component objects, and this is the basis for linking parameters between samples for co-refinement. For a comprehensive demonstration of multiple-contrast co-refinement, see the annotated notebook in the supporting information.

### 2.3. Statistical comparison and model refinement   

The Objective statistics are used directly by the CurveFitter class to perform least-squares fitting with the functionality provided by the *SciPy* package (Differential Evolution, Levenberg–Marquardt, LBFGSB – limited Broyden–Fletcher–Goldfarb–Shanno with bounds). Additional *SciPy* solvers can be added relatively simply and it would be possible for other minimizers to use Objective directly. CurveFitter can also perform Bayesian Markov-chain Monte Carlo (MCMC) sampling of the system, examining the posterior probability distribution of the parameters [equation (1)]. The posterior distribution is proportional to the product of the prior probability and the likelihood (or the sum of the log-probabilities):
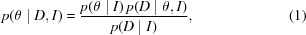


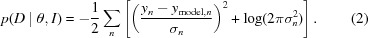
The prior, *p*(θ | *I*), is the probability distribution function for a parameter, θ, given pre-existing knowledge of the system, *I*, as outlined above. The likelihood [equation (2)], *p*(*D* | θ, *I*), is the probability of the observed data, *D*, given the model parameters and other prior information. It is calculated from the measured data, *y_n_* (with uncertainties σ_*n*_), and the generative model, *y*_model,*n*_. The likelihoods that are used here assume that the measurement uncertainties are normally distributed [equation (2)]. However, other types of measurement uncertainties (*e.g.* Poissonian) could be implemented by a subclass of Objective, overriding the log-likelihood method. The model evidence, *p*(*D* | *I*), is a normalizing factor.

The posterior probability, *p*(θ | *D*, *I*), describes the distribution of parameter values consistent with the data and prior information. In the simplest form, this is akin to a confidence interval for a parameter derived by least-squares analysis. However, when parameters are correlated, or two models give a similar quality of fit (‘multi-modality’), a simple confidence interval can be misleading. The posterior probability is derived by encoding the likelihood and prior distributions and then using an MCMC algorithm (via the *emcee* and *ptemcee* packages) to perform affine-invariant ensemble sampling (Foreman-Mackey *et al.*, 2013 ▸; Vousden *et al.*, 2016 ▸). At the end of an MCMC run, the parameter set possesses a number of samples (called a ‘chain’); the samples reveal the distribution and covariance of the parameters, the spread of the model-predicted measurements around the data and, in a reflectometry context, the range of SLD profiles that are consistent with the data. The chain statistics are used to update each Parameter value and assign a standard uncertainty. For the sampling, these represent the median and half of the [15.87, 84.13] percentile range, respectively; the latter approximates the standard deviation for a normally distributed statistic.

The *ptemcee* package is a variant (a ‘fork’ in open-source software development terms) of the *emcee* package that has been extended to implement the parallel-tempering algorithm for the characterization of multi-modal probability distributions; different modes can be traversed by chain populations at higher ‘temperatures’, while individual modes are efficiently explored by chains at lower ‘temperatures’ (Vousden *et al.*, 2016 ▸). Having multiple populations in the parallel-tempering algorithm allows the sampler to escape local maxima, greatly aiding its ability to explore the most probable regions of the posterior. *ptemcee* is also able to estimate the log-evidence term [the denominator in equation (2)], which is useful when calculating the Bayes factor for model comparison.

Parallelization of the sampling is automatic, making full use of multi-core machines, and can use MPI (message passing interface) on a cluster for yet greater parallelization. Visualization of the samples produced by MCMC sampling is performed using the *corner* package for scatter-plot matrices (Foreman-Mackey, 2016 ▸), which gives a representation of the probability distribution function for each individual parameter and also the covariance for each pair of parameters. As will be seen later, the plot for two normally distributed and uncorrelated parameters is isotropic, while covariant parameters show significant anisotropy. An evaluation of the impact of hard bounds can also be made by looking for plots where the bounds are clearly truncating the distribution function, allowing the bounds to be re-evaluated and adjusted if necessary.

### 2.4. User interface   

**Figure 2:**
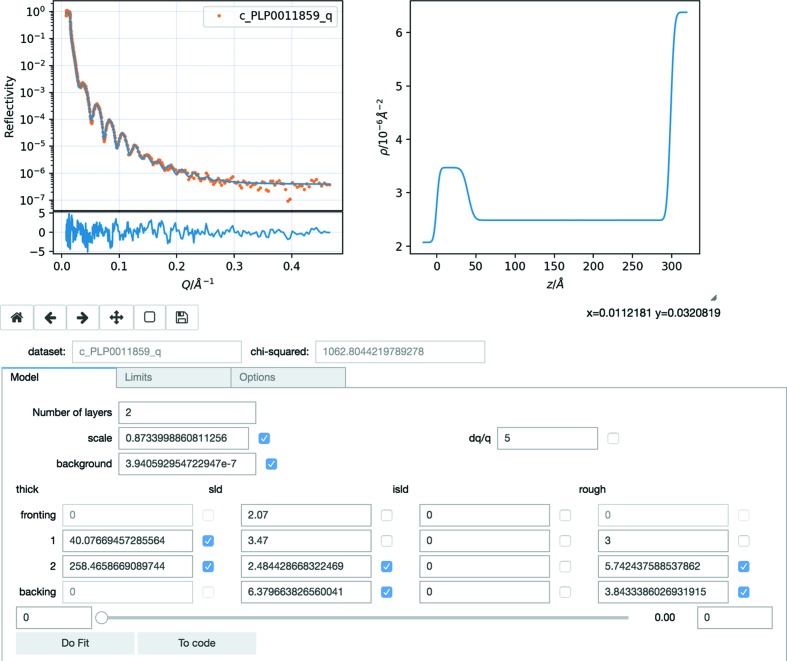
A screenshot of the *Jupyter*/*ipywidgets* GUI; this *Jupyter* notebook is available in the supporting information.

A significant motivation in the development of *refnx* has been the facilitation of reproducible analysis by helping the user describe how an analysis was performed. A few lines of computer code provide an incredibly powerful description, conveying the details with a precision that is hard to match in written text, as well as being extremely concise. Example analyses within the *refnx* code base are often sufficient to complete the task. These few lines of Python code can be further extended to produce publication-quality plots, saved and ready to import into the next publication, or to be used in a loop for batch-fitting purposes. While Python is a popular language for instruction and data analysis, meaning that the relatively few lines of code required to complete a *refnx* analysis of a set of experiments is not a huge hurdle, a simpler graphical user interface (GUI) is also provided. The browser-based GUI is available for fitting within a *Jupyter* notebook (Fig. 2 ▸), making use of the *ipywidgets* modules (Project Jupyter Contributors, 2015 ▸). The GUI has a ‘To code’ button that turns the current model into the few lines of code required to perform the analysis without using the GUI, thus providing the desired instructions for a reproducible analysis. The ability to generate analysis code also provides a stepping point for building more advanced models independently. The current GUI is able to use slab-based models for fitting a single data set, and a fully functional web-based reflectometry analysis notebook is currently available (Nelson & Prescott, 2018*a* ▸). If desired, it is possible to execute *Jupyter* notebooks in batch mode or to run the generated Python code within a Python program to complete batch-mode fitting of larger data sets. The *refnx* repository contains a growing set of examples of different uses of the *refnx* package.

## 3. Example data analysis with a lipid bilayer   

Neutron reflectometry is an ideal technique for the study of biologically relevant lipid membrane mimics and their interactions with proteins *etc*. Multiple contrast-variation measurements are necessary to reduce modelling ambiguity (due to loss of phase information in the scattering experiment) and improve the ability to determine the structure of various components in the system. The gold-standard approach for analysis of these data sets is co-refinement with a common model, and to parameterize the model in terms of chemically relevant parameters, such as the area per molecule (Campbell *et al.*, 2018 ▸). Sometimes a patchy coverage (distinct to low area per molecule) necessitates the use of an (incoherent) sum of reflectivities from different areas. *refnx* has functionality for all these requirements, such as the LipidLeaflet component for describing the head and tail groups of a lipid leaflet, and MixedReflectModel to account for patchiness.

The parameters used in the LipidLeaflet component are area per molecule (*A*), thicknesses for each of the head and tail regions (*t_x_*), sums of scattering lengths of the head and tail regions (*b_x_*), partial volumes of the head and tail groups (*V_x_*), roughness between head and tail region, and SLDs of the solvents for the head and tail groups (ρ_*x*,solv_). The overall SLDs of each of the head and tail group regions are given by
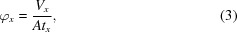


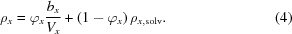
The approach used in the LipidLeaflet component ensures that there is a 1:1 correspondence of heads to tails. By default, the head and tail solvents are assumed to be the same as the solvent that is used throughout the Structure. This will be the case when using LipidLeaflet for a solid–liquid reflectometry experiment. However, at the air–liquid or liquid–liquid interfaces the solvents for the head and tail regions may be different, and it is possible to use different solvent SLDs for each. We note that the LipidLeaflet component may also be used to describe other amphiphiles adsorbing at an interface.

**Figure 3:**
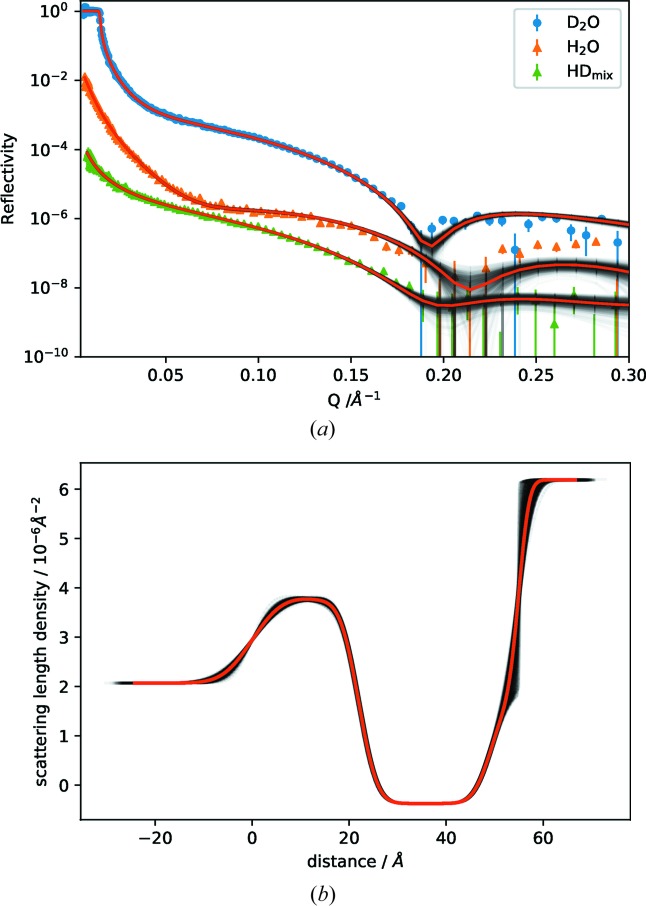
(*a*) Neutron reflectivity from a DMPC bilayer supported on a silicon crystal, measured at three contrasts, with 500 samples from the posterior distribution in grey and the median of the distribution in red. Data for the contrast-matched (HD_mix_) and H_2_O contrast are offset by 0.1 and 0.01, respectively. (*b*) The SLD profile of the D_2_O, model showing 500 samples from the posterior distribution, with the median in red. It is seen that the uncertainty in the reflectivity at high *Q* is associated with an uncertainty in the SLD profile at the lipid–D_2_O interface.

Here, LipidLeaflet is used to co-refine three contrasts [D_2_O, Si contrast-match (HD_mix_, SLD = 2.07 × 10^−6^ Å^−1^) and H_2_O] of a 1,2-dimyristoyl-*sn*-glycero-3-phosphocholine (DMPC) bilayer at the solid–liquid interface (Fig. 3 ▸). [The validity of LipidLeaflet does depend on the area per molecule being equal for the headgroup and tailgroup regions, as pointed out by Gerelli (2016 ▸), which can be violated if there are guest molecules that insert into the membrane.] Two LipidLeaflet objects are required to describe the inner and outer leaflets of a bilayer; hence the component contains an attribute which can reverse the direction of one of the leaflets. The use of individual objects to describe each leaflet leads to great flexibility: it becomes easy to model asymmetric bilayers (the inner leaflet can be a different lipid from the outer lipid), and one can model interstitial water layers between the leaflets as well.

**Figure 4:**
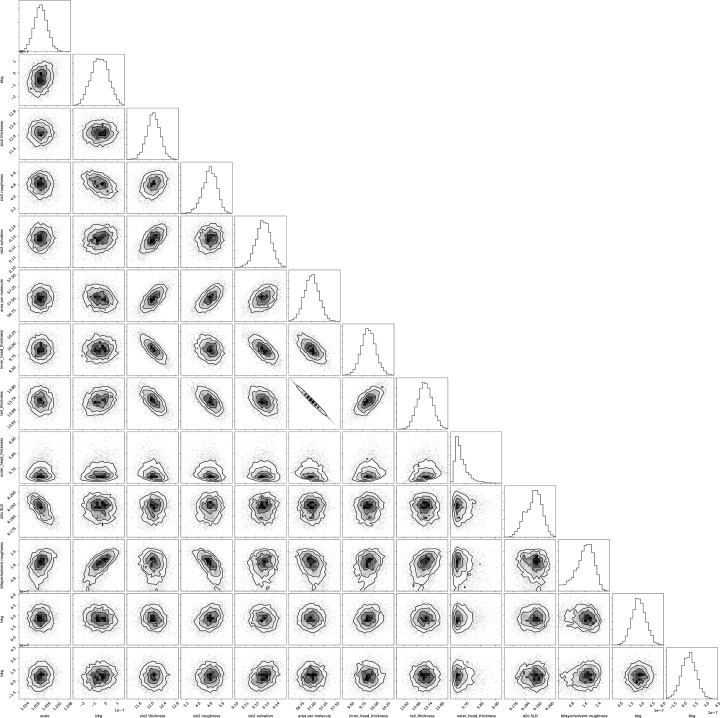
A corner plot for the varying parameters of DMPC bilayers supported on a silicon crystal, measured at three contrasts. The sampling took ∼33 min on a 2.8 GHz quad-core computer for 20 saved steps, corresponding to 4000 samples, with the steps being thinned by a factor of 400. A larger-scale image is available in the supporting information.

The *Jupyter* notebook used for the analysis, lipid.ipynb, is available in the supporting information. The corner plot (Fig. 4 ▸) produced from the MCMC analysis shows the covariance between parameters, with an area per molecule of 57.00 ± 0.15 Å. Fig. 3 ▸ shows the probability distribution of the generative model around the data and in the SLD profile. These are families of plausible fits that are obtained by plotting a subset of samples from the MCMC chain. The spread in the SLD profiles is used to determine what range of structures is consistent with the data. Multi-modalities in these SLD profiles can be due to statistical uncertainties, the *Q* ranges measured and the loss of phase information in neutron reflectometry (Majkrzak, 1999 ▸; Heinrich *et al.*, 2009 ▸).

## 4. Distribution and modification   

Each submodule in *refnx* possesses its own unit testing code for checking that the functions and classes in the module operate correctly, both individually and collectively. For example, there are tests that check that the reflectivity of a model is calculated correctly, or that the behaviour of a function is correct for the different possible inputs and code paths through it. Since the test suite is an integral part of the package, each installation is testable. In addition, there is a benchmarking suite to track changes in performance, specifically the speed of critical calculations, over time. This development approach is important for providing assurances to the community that the code is tested and works.

The source code for *refnx* is held in a publicly accessible version-controlled git repository (Nelson & Prescott, 2018*b* ▸). User contributions may be made using the standard GitHub workflow, in which contributors create their own ‘fork’ of the main *refnx* repository and create a feature branch to which they make modifications. They then submit a pull request (PR) against the main repository. The modifications made in the PR are checked on continuous-integration (CI) web services that run the test suite against a matrix of Python versions on the macOS, Linux and Windows operating systems. Features are merged into the main repository if all tests pass, and if manual code review concludes that the changes are scientifically correct, of sufficiently high standard and useful. When a sufficient number of features have accumulated, a new release is made. Successive releases have an incrementing semantic version number which can be obtained from the installed package, with each release being given its own digital object identifier (DOI). We encourage users to submit models for inclusion in a user-contributed models repository (refnx-models, https://github.com/refnx/refnx-models). We will work with users to develop a suitable way of documenting and sharing their models.

The recommended way of using *refnx* is from a *conda* environment, which offers package, dependency and environment management (Continuum Analytics, 2017 ▸), using the pre-compiled distributables on the *refnx* conda-forge channel. These distributables are made as part of the release process using the same CI web services as are used to test the code. The matrix of distributables covers the major Python versions currently in use, across the macOS, Windows and Linux operating systems. Alternatively the package can be installed from source, either directly from the git repository or via *pip* from the version uploaded to PyPI (https://pypi.python.org/pypi/refnx; the installation command is pip install refnx). Building from source requires a C compiler and the *Cython* (https://cython.org/) and *NumPy* (https://www.numpy.org/) packages to be installed; further dependencies should be installed to run the test suite to verify that compilation and installation have been successful.

*refnx* is released under the BSD permissive open-source licence. In addition, all of the dependencies of *refnx* are released under open-source licences, which means that use is free of cost to the end user and, more importantly, the user is free to modify, improve and inspect this software.

## 5. Comments on reproducibility of analyses   

In order for a given scattering analysis to be fully reproducible by others, a general set of conditions need to be met (Helliwell *et al.*, 2017 ▸; Möller *et al.*, 2017 ▸):

(i) The processed data sets used in the analysis need to be deposited with a journal article, or be freely available. Ideally, the raw data sets, and the means to create the processed data sets, should also be made available.

(ii) The exact software environment needs to be recreatable.

(iii) The exact ordered set of steps taken during the analysis needs to be listed.

Each of these points is often inadequately addressed in the literature. For example, the use of different software versions may change the output of an analysis, or the use of a GUI program may preclude recording the full set of steps, or options, applied by a user (Chirigati *et al.*, 2013 ▸). Whilst it is unable to meet the first criterion by itself, the use of *refnx* in a *Jupyter* notebook can fulfil the other two requirements, providing a little care is taken. As we have already noted, the ordered set of steps to perform the analysis is the *Jupyter* notebook in which the analysis was performed, and this is an artefact that can be archived.

The exact software environment can be recreated by noting down the versions of the software packages used during an analysis (*refnx*, *SciPy*, *NumPy*, Python *etc.*). At a later date, those exact versions can be installed in the same Python version using one of the following: the *conda* package manager, by installing from the source at a given version tag in the git repository, or by *pip*. The *conda* package manager can use environment files to recreate a specific setup. An alternative way of recreating the environment is by using a virtual machine, or another container environment such as Docker. The strengths and weaknesses of various software distribution practices and their relationship with reproducible science have been discussed in detail elsewhere (Möller *et al.*, 2017 ▸).

The usefulness of open-source software in a git (or other version-controlled) repository must be emphasized here (Möller *et al.*, 2017 ▸). With closed-source or proprietary software, the ability to return to a specific software version/environment can be frustrated, and different versions can have modifications that can unknowingly change the output of an analysis. In addition, reduced accessibility (due to cost *etc.*) to the wider scientific community can also hinder reproducibility.

Moreover, there are important ramifications for verifiability (Chirigati *et al.*, 2013 ▸). *refnx* is based on a fully open software stack, with good unit test coverage. The user can run tests for each component and inspect parts for correctness. For example, the behaviour of the reflectivity calculation in *refnx* is checked from first principles in the test suite, and this can be done now and in several years’ time. If problems are discovered, they can be corrected. With a fully or partially closed-source program, such checking is much harder, as one does not possess full knowledge of what happens inside.

## 6. Conclusions   

*refnx* is a powerful tool for least-squares or Bayesian analysis of neutron and X-ray reflectometry data that is ideally usable for reproducible research with *Jupyter* notebooks, and it has been built with extensibility in mind. Its features include MCMC sampling of posterior distribution for parameters, structural models constructed from modular components with physically relevant parameterization, algebraic inter-parameter constraints, mixed-area models, co-refinement of multiple data sets, probability distributions for parameter bounds used directly for log-prior terms and a (*Jupyter*) *ipywidgets* GUI.

### 6.1. Supporting information   

The files available in the supporting information for this article are as follows:

(i) gui.ipynb – the *Jupyter* notebook used to create the GUI screenshot.

(ii) lipid.ipynb – the *Jupyter* notebook used for the lipid analysis example.

(iii) rg5158sup2.pdf – a PDF view of the *Jupyter* notebook used for the lipid analysis example.

(iv) rg5158sup3.pdf – a larger-scale image of the corner plot shown in Fig. 4 ▸.

(v) rg5158sup4.zip – containing example lipid data sets c_PLP0016596.dat, c_PLP0016601.dat and c_PLP0016607.dat.

(vi) reduction.ipynb – the notebook for reducing example data sets.

(vii) rg5158sup5.zip – raw files for the example data sets.

(viii) refnx-paper.yml – the *conda* environment file to reproduce the analysis environment in this paper.

## Supplementary Material

Jupyter notebooks required for reproducing the analyses and figures, and refnx-paper.yml (conda environment file to reproduce the analysis environment in this paper). DOI: 10.1107/S1600576718017296/rg5158sup1.zip

PDF view of the Jupyter notebook used for the lipid analysis example. DOI: 10.1107/S1600576718017296/rg5158sup2.pdf

Larger-scale image of the corner plot. DOI: 10.1107/S1600576718017296/rg5158sup3.pdf

Example input data (c_PLP0016596.dat, c_PLP0016607.dat and c_PLP0016607.dat). DOI: 10.1107/S1600576718017296/rg5158sup4.zip

Raw files for the example data sets. DOI: 10.1107/S1600576718017296/rg5158sup5.zip
