# Supplementary material for: refnx: neutron and X-ray reflectometry analysis in Python
Source: J Appl Crystallogr. 2019 Feb 1;52(Pt 1):193–200. doi: 10.1107/S1600576718017296 (PMC6362611; doi:10.1107/S1600576718017296)

lipid

August 15, 2018

1 Co-refinement of multiple contrast DMPC datasets in *refnx*

This Jupyter notebook demonstrates the utility of the *refnx* package for analysis of neutron reflectometry data. Specifically:

- the co-refinement of three contrast variation datasets of a DMPC (1,2-dimyristoyl-sn-glycero-3-phosphocholine) bilayer measured at the solid-liquid interface with a common model
- the use of the `LipidLeaflet` component to parameterise the model in terms of physically relevant parameters
- the use of Bayesian Markov Chain Monte Carlo (MCMC) to investigate the Posterior distribution of the curvefitting system.
- the intrinsic usefulness of Jupyter notebooks to facilitate reproducible research in scientific data analysis

The images produced in this notebook are used directly in production of the *refnx* paper.

The Jupyter notebook are executable documents that can be distributed, enabling others to reproduce the data analysis contained in the document. The *refnx* documentation at https://refnx.readthedocs.io/en/latest/index.html can be consulted for further details.

The first step in most Python scripts is to import modules and functions that are going to be used

```
In [1]: # use matplotlib for plotting
        %matplotlib inline
        import matplotlib.pyplot as plt
        import numpy as np
        import os.path

        import refnx, scipy

        # the analysis module contains the curvefitting engine
        from refnx.analysis import CurveFitter, Objective, Parameter, GlobalObjective, process_

        # the reflect module contains functionality relevant to reflectometry
        from refnx.reflect import SLD, ReflectModel, Structure, LipidLeaflet

        # the ReflectDataset object will contain the data
        from refnx.dataset import ReflectDataset
```

In order for the analysis to be exactly reproducible the same package versions must be used. The *conda* packaging manager, and *pip*, can be used to ensure this is the case.

```
In [2]: # version numbers used in this analysis
        refnx.version.version, scipy.version.version
```

```
Out[2]: ('0.1.0', '1.1.0')
```

The `ReflectDataset` class is used to represent a dataset. They can be constructed by supplying a filename

```
In [3]: pth = os.path.join(os.path.dirname(refnx.__file__), 'analysis', 'test')

        data_d2o = ReflectDataset('c_PLP0016596.dat')
        data_d2o.name = "d2o"

        data_hdmix = ReflectDataset('c_PLP0016601.dat')
        data_hdmix.name = "hdmix"

        data_h2o = ReflectDataset('c_PLP0016607.dat')
        data_h2o.name = "h2o"
```

A `SLD` object is used to represent the Scattering Length Density of a material. It has `real` and `imag` attributes because the SLD is a complex number, with the imaginary part accounting for absorption. The units of SLD are $10^{-6} \mathring{A}^{-2}$

The `real` and `imag` attributes are `Parameter` objects. These `Parameter` objects contain the: parameter value, whether it allowed to vary, any interparameter constraints, and bounds applied to the parameter. The bounds applied to a parameter are probability distributions which encode the log-prior probability of the parameter having a certain value.

```
In [4]: si = SLD(2.07 + 0j)
        sio2 = SLD(3.47 + 0j)

        # the following represent the solvent contrasts used in the experiment
        d2o = SLD(6.36 + 0j)
        h2o = SLD(-0.56 + 0j)
        hdmix = SLD(2.07 + 0j)

        # We want the `real` attribute parameter to vary in the analysis, and we want to apply
        # uniform bounds. The `setp` method of a Parameter is a way of changing many aspects o
        # Parameter behaviour at once.
        d2o.real.setp(vary=True, bounds=(6.1, 6.36))
        d2o.real.name='d2o SLD'
```

The `LipidLeaflet` class is used to describe a single lipid leaflet in our interfacial model. A leaflet consists of a head and tail group region. Since we are studying a bilayer then inner and outer `LipidLeaflet`'s are required.

In [5]:
```
# Parameter for the area per molecule each DMPC molecule occupies at the surface. We
# use the same area per molecule for the inner and outer leaflets.
apm = Parameter(56, 'area per molecule', vary=True, bounds=(52, 65))

# the sum of scattering lengths for the lipid head and tail in Angstrom.
b_heads = Parameter(6.01e-4, 'b_heads')
b_tails = Parameter(-2.92e-4, 'b_tails')

# the volume occupied by the head and tail groups in cubic Angstrom.
v_heads = Parameter(319, 'v_heads')
v_tails = Parameter(782, 'v_tails')

# the head and tail group thicknesses.
inner_head_thickness = Parameter(9, 'inner_head_thickness', vary=True, bounds=(4, 11))
outer_head_thickness = Parameter(9, 'outer_head_thickness', vary=True, bounds=(4, 11))
tail_thickness = Parameter(14, 'tail_thickness', vary=True, bounds=(10, 17))

# finally construct a `LipidLeaflet` object for the inner and outer leaflets.
# Note that here the inner and outer leaflets use the same area per molecule,
# same tail thickness, etc, but this is not necessary if the inner and outer
# leaflets are different.
inner_leaflet = LipidLeaflet(apm,
                             b_heads, v_heads, inner_head_thickness,
                             b_tails, v_tails, tail_thickness,
                             3, 3)

# we reverse the monolayer for the outer leaflet because the tail groups face upwards
outer_leaflet = LipidLeaflet(apm,
                             b_heads, v_heads, outer_head_thickness,
                             b_tails, v_tails, tail_thickness,
                             3, 0, reverse_monolayer=True)
```

The `Slab` Component represents a layer of uniform scattering length density of a given thickness in our interfacial model. Here we make `Slab`s from `SLD` objects, but other approaches are possible.

In [6]:
```
# Slab constructed from SLD object.
sio2_slab = sio2(15, 3)
sio2_slab.thick.setp(vary=True, bounds=(2, 30))
sio2_slab.thick.name = 'sio2 thickness'
sio2_slab.rough.setp(vary=True, bounds=(0, 7))
sio2_slab.rough.name = name='sio2 roughness'
sio2_slab.vfsolv.setp(0.1, vary=True, bounds=(0., 0.5))
sio2_slab.vfsolv.name = 'sio2 solvation'

solv_roughness = Parameter(3, 'bilayer/solvent roughness')
solv_roughness.setp(vary=True, bounds=(0, 5))
```

Once all the `Components` have been constructed we can chain them together to compose a

Structure object. The Structure object represents the interfacial structure of our system. We create different Structures for each contrast. It is important to note that each of the Structures share many components, such as the LipidLeaflet objects. This means that parameters used to construct those components are shared between all the Structures, which enables co-refinement of multiple datasets. An alternate way to carry this out would be to apply constraints to underlying parameters, but this way is clearer. Note that the final component for each structure is a Slab created from the solvent SLDs, we give those slabs a zero thickness.

```
In [7]: s_d2o = si | sio2_slab | inner_leaflet | outer_leaflet | d2o(0, solv_roughness)
        s_hdmix = si | sio2_slab | inner_leaflet | outer_leaflet | hdmix(0, solv_roughness)
        s_h2o = si | sio2_slab | inner_leaflet | outer_leaflet | h2o(0, solv_roughness)
```

The Structures created in the previous step describe the interfacial structure, these structures are used to create ReflectModel objects that know how to apply resolution smearing, scaling factors and background.

```
In [8]: model_d2o = ReflectModel(s_d2o)
        model_hdmix = ReflectModel(s_hdmix)
        model_h2o = ReflectModel(s_h2o)

        model_d2o.scale.setp(vary=True, bounds=(0.9, 1.1))

        model_d2o.bkg.setp(vary=True, bounds=(-5e-7, 1e-6))
        model_hdmix.bkg.setp(vary=True, bounds=(-5e-7, 1e-6))
        model_h2o.bkg.setp(vary=True, bounds=(-5e-7, 1e-6))
```

An Objective is constructed from a ReflectDataset and ReflectModel. Amongst other things Objectives can calculate chi-squared, log-likelihood probability, log-prior probability, etc. We then combine all the individual Objectives into a GlobalObjective.

```
In [9]: objective_d2o = Objective(model_d2o, data_d2o)
        objective_hdmix = Objective(model_hdmix, data_hdmix)
        objective_h2o = Objective(model_h2o, data_h2o)

        global_objective = GlobalObjective([objective_d2o, objective_hdmix, objective_h2o])
```

A CurveFitter object can perform least squares fitting, or MCMC sampling on the Objective used to construct it.

```
In [10]: fitter = CurveFitter(global_objective, nwalkers=200)
```

We initialise the MCMC walkers by jittering around the best fit. Other modes of initialisation are possible: from a supplied covariance matrix, by sampling from the prior distributions, or by supplying known positions from an array.

```
In [11]: # we seed the numpy random number generator to get reproducible numbers
         # during walker initialisation
         np.random.seed(1)
         fitter.initialise('jitter')
```

In MCMC sampling a burn in period is used to allow the workers to be more representative of the distribution they are sampling. Here we do a number of samples, then discard them. The last chain position is kept to provide a starting point for the 'production' run.

```
In [12]: # set random_state for reproducible pseudo-random number streams
         fitter.sample(1000, random_state=321);
```

```
100%|| 1000/1000 [04:04<00:00,  4.32it/s]
```

The shape of the chain containing the samples is `(number_steps, number_walkers, number_parameters)`

```
In [13]: print(fitter.chain.shape)
```

```
(1000, 200, 13)
```

At the start of the sampling run the walkers in the MCMC ensemble probably won't distributed according the distribution they are sampling. We can discard, or burn, the initial steps. Let's have a look at the steps for a parameter (e.g. the area-per-molecule) to see if they've reached equilibrium (i.e. distributed around a mean).

```
In [14]: for i in range(200):
             plt.plot(fitter.chain[:, i, 5].flat)
```

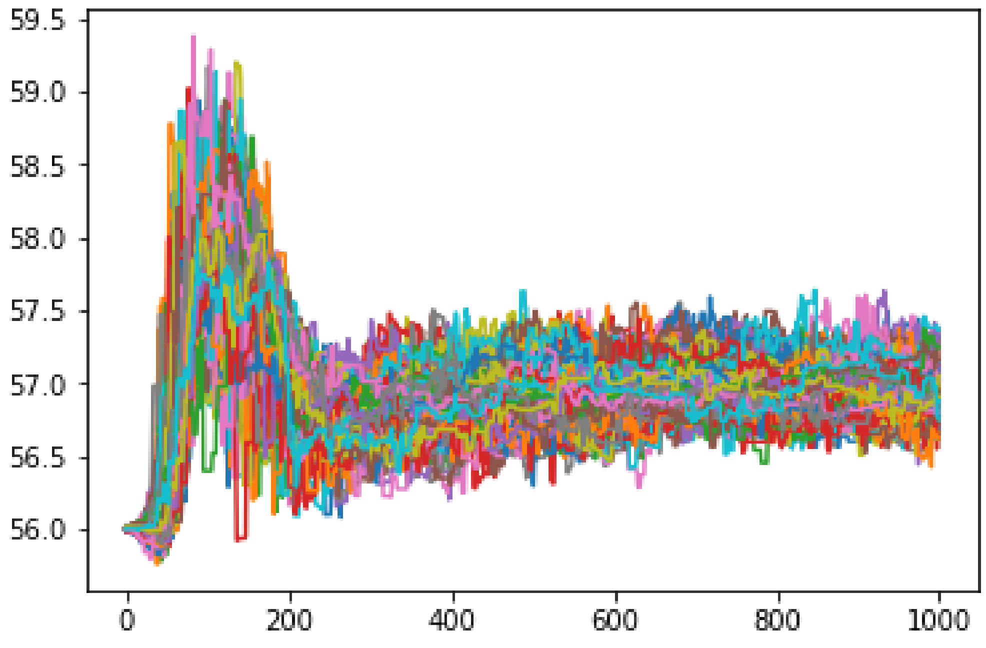

Although it's hard to tell from this graph it seems that ~500 steps is enough for equilibration, so let's discard these initial steps that acted as the burn-in period.

```
In [15]: fitter.reset()
```

Now we do a production sampling run. In this example the total number of samples is the number of walkers (200 by default) multiplied by the number of steps: 8000 * 200 = 1 600 000. The sampling engine automatically makes full use of the total number of processing cores available to it, but this is specifiable. In addition MPI can be used, which make it useful for sampling on a cluster - MCMC is embarrassingly parallel. Samples can be saved to file as they are acquired, useful for checkpointing sampling state.

```
In [16]: fitter.sample(8000, random_state=123);
```

```
100%|| 8000/8000 [31:20<00:00,  4.41it/s]
```

However, successive steps are correlated to previous steps to some degree, and the chain should be thinned to ensure the samples are independent. Let's see how much we should thin by by looking at the autocorrelation of a parameter.

```
In [17]: plt.plot(fitter.acf()[:, 5])
         plt.xlim(0, 1000);
```

```
/Users/anz/miniconda3/envs/dev3/lib/python3.6/site-packages/mkl_fft/_numpy_fft.py:158: FutureWa
  output = mkl_fft.fft(a, n, axis)
```

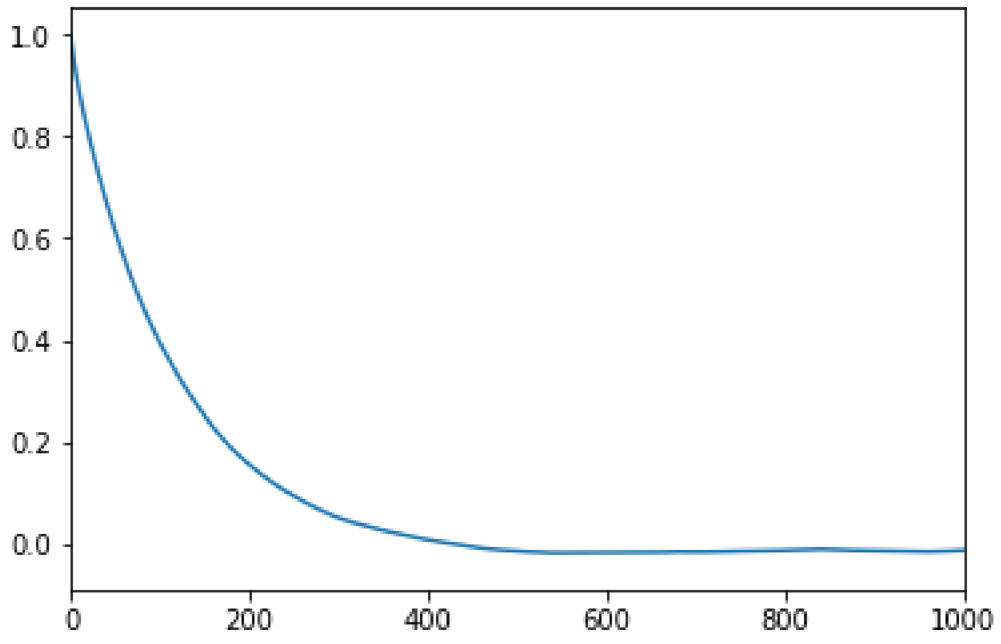

For the sampling done here thinning by 400 should be sufficient.

```
In [18]: process_chain(global_objective, fitter.chain, nthin=400);
```

The sampling gives each varying parameter its own MCMC chain, which can be processed to give relevant statistics, or histogrammed, etc. The relationship between chains encodes the covariance of all the parameters. The chains are automatically processed to calculate the median of all the samples, and the half width of the [15.87, 84.13] percentiles. These two values are taken to be the 'fitted' parameter value, and its standard deviation. Each Parameter set to this median value, and given an `stderr` attribute. We can see those statistics by printing the objective.

```
In [19]: print(global_objective)
```

```
_______________________________________________________________________________

--Global Objective--
________________________________________________________________________________
Objective - 112000576592
Dataset = d2o
datapoints = 146
chi2 = 458.2338467947417
Weighted = True
Transform = None
________________________________________________________________________________
Parameters:       ''
________________________________________________________________________________
Parameters: 'instrument parameters'
<Parameter:    'scale'    value=    1.02274     +/- 0.00358, bounds=[0.9, 1.1]>
<Parameter:     'bkg'     value= -4.94791e-08   +/- 7.39e-08, bounds=[-5e-07, 1e-06]>
<Parameter:'dq - resolution'value=       5        (fixed)  , bounds=[-inf, inf]>
________________________________________________________________________________
Parameters: 'Structure - '
________________________________________________________________________________
Parameters:       ''
<Parameter:  ' - thick'   value=       0        (fixed)  , bounds=[-inf, inf]>
<Parameter:   ' - sld'    value=     2.07       (fixed)  , bounds=[-inf, inf]>
<Parameter:   ' - isld'   value=       0        (fixed)  , bounds=[-inf, inf]>
<Parameter:  ' - rough'   value=       0        (fixed)  , bounds=[-inf, inf]>
<Parameter:' - volfrac solvent'value=       0        (fixed)  , bounds=[-inf, inf]>
________________________________________________________________________________
Parameters:       ''
<Parameter:'sio2 thickness'value=    12.0885     +/- 0.215, bounds=[2, 30]>
<Parameter:   ' - sld'    value=     3.47       (fixed)  , bounds=[-inf, inf]>
<Parameter:   ' - isld'   value=       0        (fixed)  , bounds=[-inf, inf]>
<Parameter:'sio2 roughness'value=    4.80879     +/- 0.478, bounds=[0, 7]>
<Parameter:'sio2 solvation'value=   0.127675     +/- 0.00728, bounds=[0.0, 0.5]>
________________________________________________________________________________
Parameters:       ''
<Parameter:'area per molecule'value=    56.9956     +/- 0.156, bounds=[52, 65]>
<Parameter:   'b_heads'   value=   0.000601     (fixed)  , bounds=[-inf, inf]>
<Parameter:' - b_heads_imag'value=       0        (fixed)  , bounds=[-inf, inf]>
<Parameter:   'v_heads'   value=      319       (fixed)  , bounds=[-inf, inf]>
```

```
<Parameter:'inner_head_thickness'value=    9.86971     +/- 0.151, bounds=[4, 11]>
<Parameter:   'b_tails'   value=   -0.000292    (fixed)  , bounds=[-inf, inf]>
<Parameter:' - b_tails_imag'value=       0        (fixed)  , bounds=[-inf, inf]>
<Parameter:   'v_tails'   value=      782       (fixed)  , bounds=[-inf, inf]>
<Parameter:'tail_thickness'value=    13.7233     +/- 0.0373, bounds=[10, 17]>
<Parameter:' - rough_head_tail'value=       3        (fixed)  , bounds=[-inf, inf]>
<Parameter:' - rough_fronting_mono'value=       3        (fixed)  , bounds=[-inf, inf]>
--------------------------------------------------------------------------------
Parameters:       ''
<Parameter:'area per molecule'value=    56.9956     +/- 0.156, bounds=[52, 65]>
<Parameter:   'b_heads'   value=   0.000601     (fixed)  , bounds=[-inf, inf]>
<Parameter:' - b_heads_imag'value=       0        (fixed)  , bounds=[-inf, inf]>
<Parameter:   'v_heads'   value=      319       (fixed)  , bounds=[-inf, inf]>
<Parameter:'outer_head_thickness'value=    5.64468     +/- 0.0547, bounds=[4, 11]>
<Parameter:   'b_tails'   value=   -0.000292    (fixed)  , bounds=[-inf, inf]>
<Parameter:' - b_tails_imag'value=       0        (fixed)  , bounds=[-inf, inf]>
<Parameter:   'v_tails'   value=      782       (fixed)  , bounds=[-inf, inf]>
<Parameter:'tail_thickness'value=    13.7233     +/- 0.0373, bounds=[10, 17]>
<Parameter:' - rough_head_tail'value=       3        (fixed)  , bounds=[-inf, inf]>
<Parameter:' - rough_fronting_mono'value=       0        (fixed)  , bounds=[-inf, inf]>
--------------------------------------------------------------------------------
Parameters:       ''
<Parameter:  ' - thick'   value=       0        (fixed)  , bounds=[-inf, inf]>
<Parameter:   'd2o SLD'   value=    6.18989     +/- 0.00559, bounds=[6.1, 6.36]>
<Parameter:   ' - isld'   value=       0        (fixed)  , bounds=[-inf, inf]>
<Parameter:'bilayer/solvent roughness'value=    1.64969     +/- 0.554, bounds=[0, 5]>
<Parameter:' - volfrac solvent'value=       0        (fixed)  , bounds=[-inf, inf]>


--------------------------------------------------------------------------------
Objective - 112000575864
Dataset = hdmix
datapoints = 97
chi2 = 110.57212193739815
Weighted = True
Transform = None
--------------------------------------------------------------------------------
Parameters:       ''
--------------------------------------------------------------------------------
Parameters: 'instrument parameters'
<Parameter:    'scale'    value=       1        (fixed)  , bounds=[-inf, inf]>
<Parameter:     'bkg'     value=  2.60714e-07   +/- 9.78e-08, bounds=[-5e-07, 1e-06]>
<Parameter:'dq - resolution'value=       5        (fixed)  , bounds=[-inf, inf]>
--------------------------------------------------------------------------------
Parameters: 'Structure - '
--------------------------------------------------------------------------------
Parameters:       ''
<Parameter:  ' - thick'   value=       0        (fixed)  , bounds=[-inf, inf]>
```

```
<Parameter:   ' - sld'    value=     2.07       (fixed)  , bounds=[-inf, inf]>
<Parameter:   ' - isld'   value=       0        (fixed)  , bounds=[-inf, inf]>
<Parameter:  ' - rough'   value=       0        (fixed)  , bounds=[-inf, inf]>
<Parameter:' - volfrac solvent'value=       0        (fixed)  , bounds=[-inf, inf]>
--------------------------------------------------------------------------------
Parameters:       ''
<Parameter:'sio2 thickness'value=    12.0885     +/- 0.215, bounds=[2, 30]>
<Parameter:   ' - sld'    value=     3.47       (fixed)  , bounds=[-inf, inf]>
<Parameter:   ' - isld'   value=       0        (fixed)  , bounds=[-inf, inf]>
<Parameter:'sio2 roughness'value=    4.80879     +/- 0.478, bounds=[0, 7]>
<Parameter:'sio2 solvation'value=   0.127675     +/- 0.00728, bounds=[0.0, 0.5]>
--------------------------------------------------------------------------------
Parameters:       ''
<Parameter:'area per molecule'value=    56.9956     +/- 0.156, bounds=[52, 65]>
<Parameter:   'b_heads'   value=   0.000601     (fixed)  , bounds=[-inf, inf]>
<Parameter:' - b_heads_imag'value=       0        (fixed)  , bounds=[-inf, inf]>
<Parameter:   'v_heads'   value=      319       (fixed)  , bounds=[-inf, inf]>
<Parameter:'inner_head_thickness'value=    9.86971     +/- 0.151, bounds=[4, 11]>
<Parameter:   'b_tails'   value=   -0.000292    (fixed)  , bounds=[-inf, inf]>
<Parameter:' - b_tails_imag'value=       0        (fixed)  , bounds=[-inf, inf]>
<Parameter:   'v_tails'   value=      782       (fixed)  , bounds=[-inf, inf]>
<Parameter:'tail_thickness'value=    13.7233     +/- 0.0373, bounds=[10, 17]>
<Parameter:' - rough_head_tail'value=       3        (fixed)  , bounds=[-inf, inf]>
<Parameter:' - rough_fronting_mono'value=       3        (fixed)  , bounds=[-inf, inf]>
--------------------------------------------------------------------------------
Parameters:       ''
<Parameter:'area per molecule'value=    56.9956     +/- 0.156, bounds=[52, 65]>
<Parameter:   'b_heads'   value=   0.000601     (fixed)  , bounds=[-inf, inf]>
<Parameter:' - b_heads_imag'value=       0        (fixed)  , bounds=[-inf, inf]>
<Parameter:   'v_heads'   value=      319       (fixed)  , bounds=[-inf, inf]>
<Parameter:'outer_head_thickness'value=    5.64468     +/- 0.0547, bounds=[4, 11]>
<Parameter:   'b_tails'   value=   -0.000292    (fixed)  , bounds=[-inf, inf]>
<Parameter:' - b_tails_imag'value=       0        (fixed)  , bounds=[-inf, inf]>
<Parameter:   'v_tails'   value=      782       (fixed)  , bounds=[-inf, inf]>
<Parameter:'tail_thickness'value=    13.7233     +/- 0.0373, bounds=[10, 17]>
<Parameter:' - rough_head_tail'value=       3        (fixed)  , bounds=[-inf, inf]>
<Parameter:' - rough_fronting_mono'value=       0        (fixed)  , bounds=[-inf, inf]>
--------------------------------------------------------------------------------
Parameters:       ''
<Parameter:  ' - thick'   value=       0        (fixed)  , bounds=[-inf, inf]>
<Parameter:   ' - sld'    value=     2.07       (fixed)  , bounds=[-inf, inf]>
<Parameter:   ' - isld'   value=       0        (fixed)  , bounds=[-inf, inf]>
<Parameter:'bilayer/solvent roughness'value=    1.64969     +/- 0.554, bounds=[0, 5]>
<Parameter:' - volfrac solvent'value=       0        (fixed)  , bounds=[-inf, inf]>


--------------------------------------------------------------------------------
Objective - 112000576088
```

```
Dataset = h2o
datapoints = 104
chi2 = 249.65438269583709
Weighted = True
Transform = None
--------------------------------------------------------------------------------
Parameters:       ''
--------------------------------------------------------------------------------
Parameters: 'instrument parameters'
<Parameter:    'scale'    value=       1        (fixed)  , bounds=[-inf, inf]>
<Parameter:     'bkg'     value=  6.16827e-08   +/- 9.75e-08, bounds=[-5e-07, 1e-06]>
<Parameter:'dq - resolution'value=       5        (fixed)  , bounds=[-inf, inf]>
--------------------------------------------------------------------------------
Parameters: 'Structure - '
--------------------------------------------------------------------------------
Parameters:       ''
<Parameter:  ' - thick'   value=       0        (fixed)  , bounds=[-inf, inf]>
<Parameter:   ' - sld'    value=     2.07       (fixed)  , bounds=[-inf, inf]>
<Parameter:   ' - isld'   value=       0        (fixed)  , bounds=[-inf, inf]>
<Parameter:  ' - rough'   value=       0        (fixed)  , bounds=[-inf, inf]>
<Parameter:' - volfrac solvent'value=       0        (fixed)  , bounds=[-inf, inf]>
--------------------------------------------------------------------------------
Parameters:       ''
<Parameter:'sio2 thickness'value=    12.0885     +/- 0.215, bounds=[2, 30]>
<Parameter:   ' - sld'    value=     3.47       (fixed)  , bounds=[-inf, inf]>
<Parameter:   ' - isld'   value=       0        (fixed)  , bounds=[-inf, inf]>
<Parameter:'sio2 roughness'value=    4.80879     +/- 0.478, bounds=[0, 7]>
<Parameter:'sio2 solvation'value=   0.127675     +/- 0.00728, bounds=[0.0, 0.5]>
--------------------------------------------------------------------------------
Parameters:       ''
<Parameter:'area per molecule'value=    56.9956     +/- 0.156, bounds=[52, 65]>
<Parameter:   'b_heads'   value=   0.000601     (fixed)  , bounds=[-inf, inf]>
<Parameter:' - b_heads_imag'value=       0        (fixed)  , bounds=[-inf, inf]>
<Parameter:   'v_heads'   value=      319       (fixed)  , bounds=[-inf, inf]>
<Parameter:'inner_head_thickness'value=    9.86971     +/- 0.151, bounds=[4, 11]>
<Parameter:   'b_tails'   value=   -0.000292    (fixed)  , bounds=[-inf, inf]>
<Parameter:' - b_tails_imag'value=       0        (fixed)  , bounds=[-inf, inf]>
<Parameter:   'v_tails'   value=      782       (fixed)  , bounds=[-inf, inf]>
<Parameter:'tail_thickness'value=    13.7233     +/- 0.0373, bounds=[10, 17]>
<Parameter:' - rough_head_tail'value=       3        (fixed)  , bounds=[-inf, inf]>
<Parameter:' - rough_fronting_mono'value=       3        (fixed)  , bounds=[-inf, inf]>
--------------------------------------------------------------------------------
Parameters:       ''
<Parameter:'area per molecule'value=    56.9956     +/- 0.156, bounds=[52, 65]>
<Parameter:   'b_heads'   value=   0.000601     (fixed)  , bounds=[-inf, inf]>
<Parameter:' - b_heads_imag'value=       0        (fixed)  , bounds=[-inf, inf]>
<Parameter:   'v_heads'   value=      319       (fixed)  , bounds=[-inf, inf]>
<Parameter:'outer_head_thickness'value=    5.64468     +/- 0.0547, bounds=[4, 11]>
```

```
<Parameter:   'b_tails'   value=   -0.000292    (fixed)   , bounds=[-inf, inf]>
<Parameter:' - b_tails_imag'value=       0        (fixed)   , bounds=[-inf, inf]>
<Parameter:   'v_tails'   value=      782       (fixed)   , bounds=[-inf, inf]>
<Parameter:'tail_thickness'value=    13.7233     +/- 0.0373, bounds=[10, 17]>
<Parameter:' - rough_head_tail'value=       3        (fixed)   , bounds=[-inf, inf]>
<Parameter:' - rough_fronting_mono'value=       0        (fixed)   , bounds=[-inf, inf]>

--------------------------------------------------------------------------------
Parameters:       ''
<Parameter:  ' - thick'   value=       0        (fixed)   , bounds=[-inf, inf]>
<Parameter:   ' - sld'    value=     -0.56      (fixed)   , bounds=[-inf, inf]>
<Parameter:   ' - isld'   value=       0        (fixed)   , bounds=[-inf, inf]>
<Parameter:'bilayer/solvent roughness'value=    1.64969     +/- 0.554, bounds=[0, 5]>
<Parameter:' - volfrac solvent'value=       0        (fixed)   , bounds=[-inf, inf]>
```

Now let's see how the 'fitted' models compare to the data. We could use `global_objective.plot()`, but because we want to do a bit more tweaking for the graphics (such as vertical offsets) we're going to create the graph manually. We're also going to examine the spread in the posterior distribution.

```
In [20]: hdmix_mult = 0.01
         h2o_mult = 0.1
         # the data
         plt.errorbar(data_d2o.x, data_d2o.y, data_d2o.y_err,
                      label='$\mathregular{D_2O}$', ms=4, marker='o', lw=0, elinewidth=1)
         plt.errorbar(data_h2o.x, data_h2o.y * h2o_mult, data_h2o.y_err * h2o_mult,
                      label='$\mathregular{H_2O}$', ms=4, marker='^', lw=0, elinewidth=1)
         plt.errorbar(data_hdmix.x, data_hdmix.y * hdmix_mult, data_hdmix.y_err * hdmix_mult,
                      label='$\mathregular{HD_{mix}}$', ms=4, marker='^', lw=0, elinewidth=1)

         # the median of the posterior
         plt.plot(data_d2o.x, objective_d2o.generative(), color='r', zorder=20)
         plt.plot(data_hdmix.x, objective_hdmix.generative() * hdmix_mult, color='r', zorder=2
         plt.plot(data_h2o.x, objective_h2o.generative() * h2o_mult, color='r', zorder=20)

         # plot the spread of the fits for the different datasets
         gen = global_objective.pgen(500)

         save_pars = np.copy(global_objective.parameters)
         for i in range(500):
             global_objective.setp(next(gen))

             plt.plot(data_d2o.x, objective_d2o.generative(),
                      color='k', alpha=0.02, zorder=10)
             plt.plot(data_hdmix.x, objective_hdmix.generative() * hdmix_mult,
                      color='k', alpha=0.02, zorder=10)
```

```
    plt.plot(data_h2o.x, objective_h2o.generative() * h2o_mult,
             color='k', alpha=0.02, zorder=10)

# put back the saved parameters
global_objective.setp(save_pars)

ax = plt.gca()
ax.text(-0.04, 1e-11, 'a)')
plt.legend()
plt.yscale('log')
plt.ylabel('Reflectivity')
plt.xlabel('Q /$\AA^{-1}$')
plt.ylim(1e-10, 2);
plt.xlim(0.004, 0.3)
plt.savefig('global_fit.pdf')
```

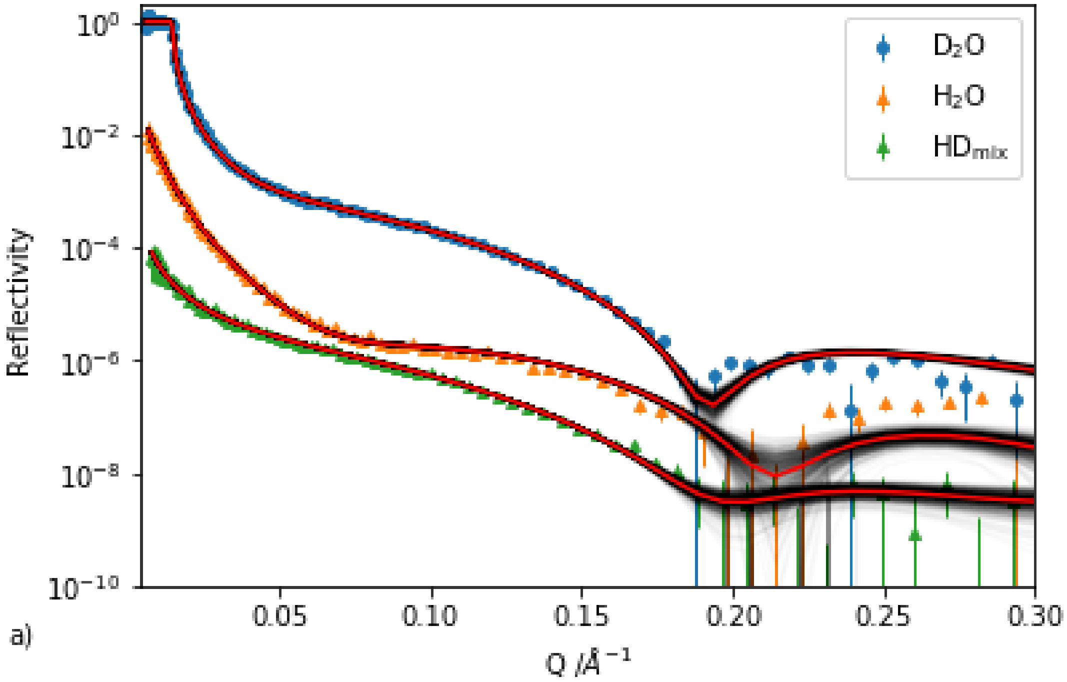

We can investigate the posterior distribution by a corner plot, this reveals interparameter covariances.

```
In [21]: global_objective.corner();
         plt.savefig('corner.pdf')
```

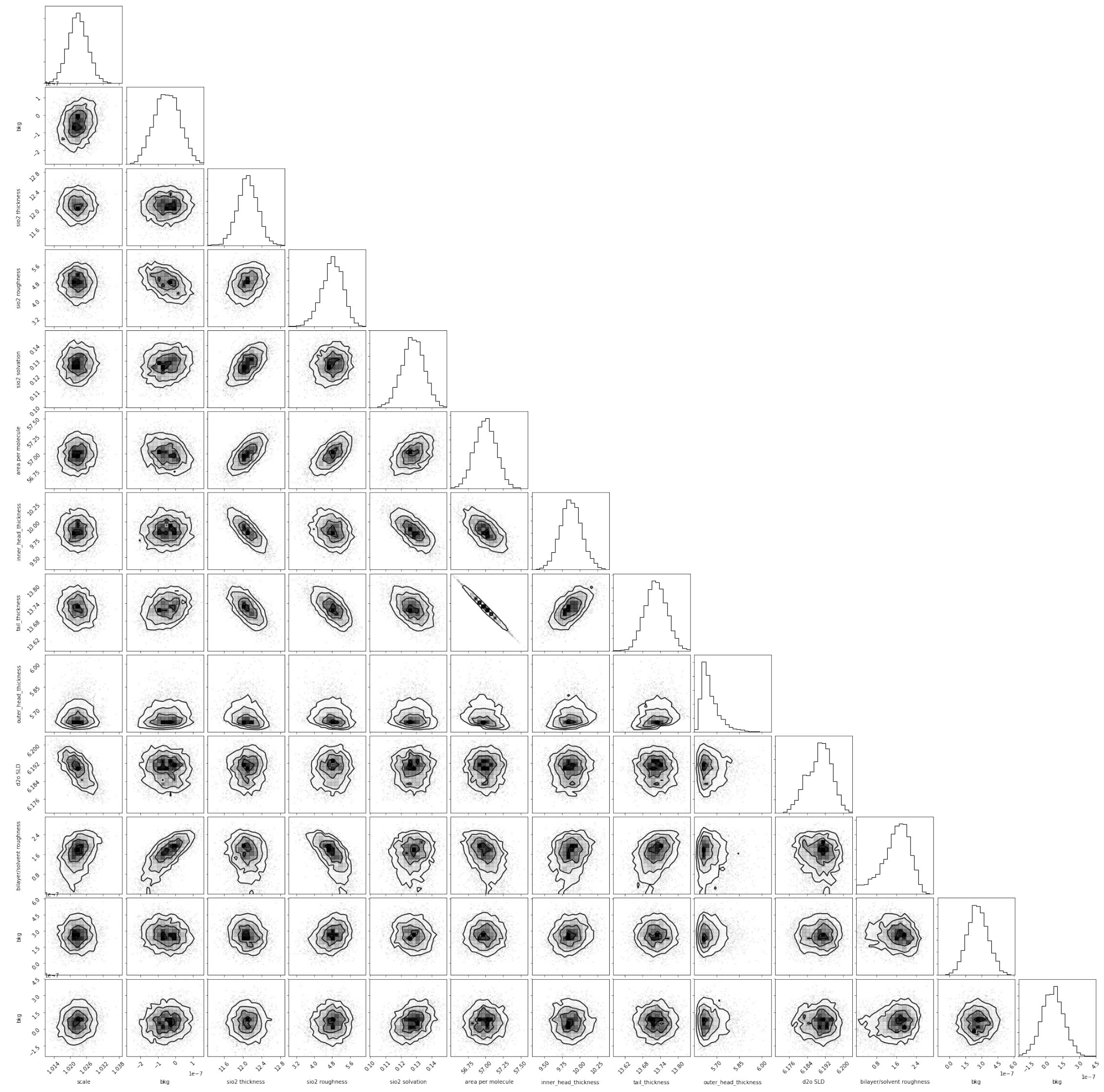

The variation in scattering length density profiles can be visualised by a little bit of processing. This enables one to see what range of SLD profiles are statistically possible.

In [22]:
```
saved_params = np.array(objective_d2o.parameters)

z, median_sld = s_d2o.sld_profile()

for pvec in objective_d2o.pgen(ngen=500):
    objective_d2o.setp(pvec)
    zs, sld = s_d2o.sld_profile()
    plt.plot(zs, sld, color='k', alpha=0.05)

# put back saved_params
objective_d2o.setp(saved_params)
```

```
ax = plt.gca()
ax.text(-50, -1.6, 'b)')
plt.plot(z, median_sld, lw=2, color='r');
plt.ylabel('scattering length density / $10^{-6}\AA^{-2}$')
plt.xlabel('distance / $\AA$')
plt.savefig('d2o_sld_spread.pdf')
```

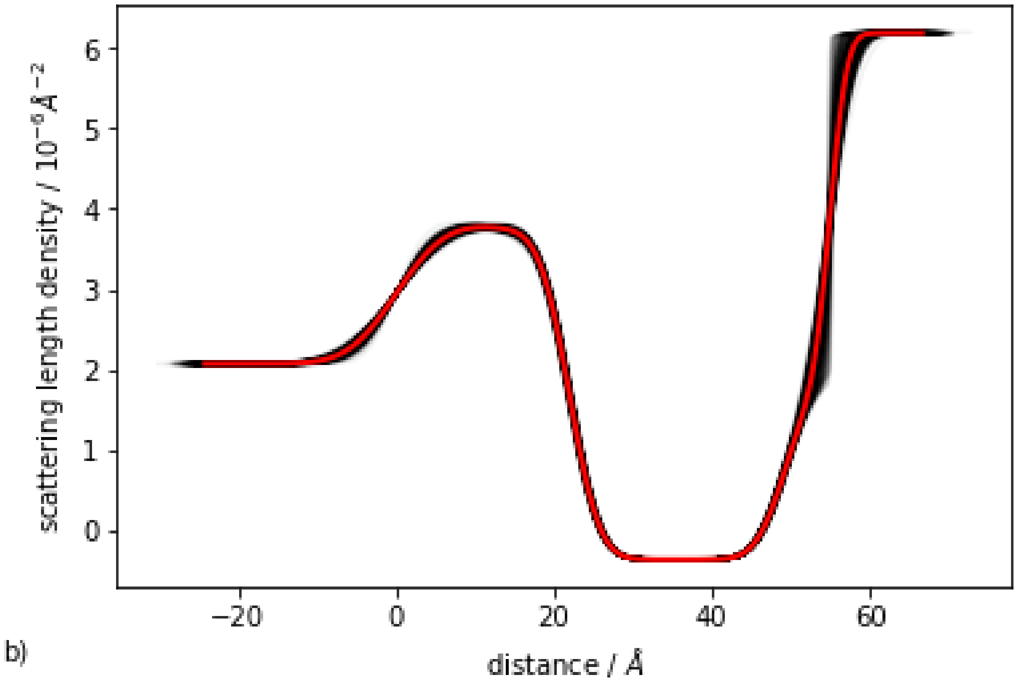

Supplement: Supplementary file 2 [file j-52-00193-sup2.pdf]
