# Supplementary figures and images for: refnx: neutron and X-ray reflectometry analysis in Python
Source: J Appl Crystallogr. 2019 Feb 1;52(Pt 1):193–200. doi: 10.1107/S1600576718017296 (PMC6362611; doi:10.1107/S1600576718017296)

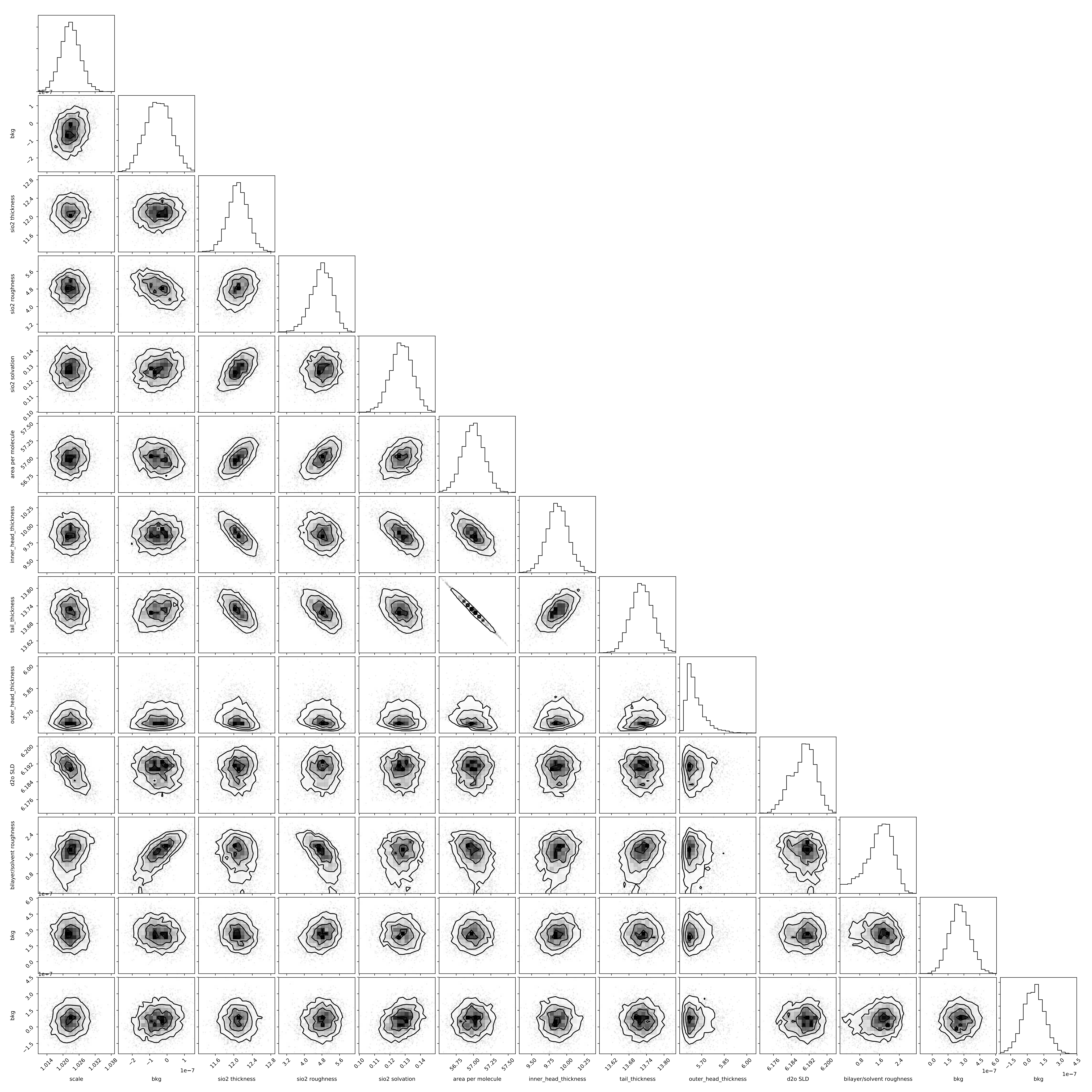

Supplement: Supplementary file 3 [file j-52-00193-sup3.pdf]
